# Genetic Predisposition Between COVID-19 and Four Mental Illnesses: A Bidirectional, Two-Sample Mendelian Randomization Study

**DOI:** 10.3389/fpsyt.2021.746276

**Published:** 2021-10-20

**Authors:** Ningning Liu, Jiang-Shan Tan, Lu Liu, Yufeng Wang, Lu Hua, Qiujin Qian

**Affiliations:** ^1^Peking University Sixth Hospital/Institute of Mental Health, Beijing, China; ^2^NHC Key Laboratory of Mental Health (Peking University), National Clinical Research Center for Mental Disorders (Peking University Sixth Hospital), Beijing, China; ^3^Thrombosis Center, Key Laboratory of Pulmonary Vascular Medicine, National Clinical Research Center of Cardiovascular Diseases, State Key Laboratory of Cardiovascular Disease and Fuwai Hospital, Chinese Academy of Medical Sciences and Peking Union Medical College, Beijing, China

**Keywords:** COVID-19, mental illness, GWAS, risk, Mendelian randomization

## Abstract

**Background:** The outbreak of 2019 coronavirus disease (COVID-19) has become a global pandemic. Although it has long been suspected that COVID-19 could contribute to the development of mental illness, and individuals with a pre-existing mental illness may have a higher risk of and poorer outcomes from COVID-19 infection, no evidence has established a causal association between them thus far.

**Methods:** To investigate associations in support of a causal association between the severity of COVID-19 and mental illnesses, we leveraged large-scale genetic summary data from genome-wide association study (GWAS) summary datasets, including attention-deficit/hyperactivity disorder (ADHD) (*n* = 55,374), schizophrenia (*n* = 77,096), bipolar disorder (*n* = 51,710), and depression (*n* = 173,005), based on a previous observational study. The random-effects inverse-variance weighted method was conducted for the main analyses, with a complementary analysis of the weighted median and MR-Egger approaches and multiple sensitivity analyses assessing horizontal pleiotropy and removing outliers in two different COVID-19 databases.

**Results:** The Mendelian randomization (MR) analysis indicated that ADHD [odds ratio (OR) = 1.297; 95% confidence interval (CI), 1.029–1.634; *p* = 0.028] increased the risk of hospitalization due to COVID-19. A similar association was obtained in MR sensitivity analyses of the weighted median. In addition, genetically predicted COVID-19 was significantly associated with schizophrenia (OR = 1.043; 95% CI, 1.005–1.082; *p* = 0.027).

**Conclusions:** Although many studies have reported a causal relationship between COVID-19 and mental illness, our study shows that this increased risk is modest. However, considering the characteristics of ADHD that might further increase the individuals' vulnerability to being infected by COVID-19, the ongoing massive worldwide exposure to COVID-19, and the high burden of schizophrenia, we believe that it is necessary to offer preventative measures to these populations and to provide more evidence in understanding the neurological impact of COVID-19.

## Introduction

Severe acute respiratory syndrome coronavirus 2 (SARS-CoV-2) or 2019 coronavirus disease (COVID-19), a new type of highly infectious coronavirus that can cause severe respiratory illness, and even death, has emerged, causing a global pandemic. As of April 29, 2021, more than 124 million people have been confirmed to be infected worldwide. Since the start of the COVID-19 pandemic, many countries have reported high rates of psychiatric symptoms in individuals infected with COVID-19 (1–5). At the same time, there are growing concerns that people with pre-existing mental illnesses may have a higher risk and poorer outcomes of COVID-19 infection (6–8).

Indeed, by clinical observations and questionnaire surveys, investigators have found that patients with COVID-19 have higher levels of neuropsychiatric complications, including anxiety disorder (6–8), depression (9), attention-deficit/hyperactivity disorder (ADHD) (10), and psychotic disorder (11). Recently, one nationwide study across 50 states in the US also found that patients with a recent diagnosis of mental illnesses had significantly increased risks of COVID-19 infection (12). Another study based on 69.8 million patients from the US suggested that survivors of COVID-19 seemed to be at increased risk of psychiatric sequelae (13). While suggestive, these studies could not provide evidence of a causal association between COVID-19 and psychotic disorders.

First, most findings are based on correlational studies, so no conclusion can yet be reached about whether individuals infected with COVID-19 would be at enhanced risk of acquiring psychosis or vice versa. Alternatively, some published studies are based on small sample sizes and could not adjust for potential confounding factors. Mental illness is a highly heterogeneous disease, and multiple individual vulnerabilities and traits could potentially contribute to its onset. Meanwhile, multiple factors may increase the severity of COVID-19, such as socioeconomic deprivation, older age, and cardiovascular disease (14–18)_._ Therefore, several specific complexities, such as social, cultural, and psychological circumstances and iatrogenic influences, need to be considered (19) when assessing the causal association between them. However, for now, it is impractical and unrealistic to consider all these potential confounding factors. Finally, due to their poor psychometric properties, traditional screening tools cannot provide accurate assessments of the cases emerging in this current pandemic. Meanwhile, the clinical utility, methodological strengths, and limitations of new scales have not yet been explored. That is, robust screening tools or diagnostic instruments that could be used during COVID-19 are still limited (20). Therefore, although the association between COVID-19 and mental illness has been reported in many observational studies, causality cannot be reliably inferred.

Mendelian randomization (MR) is a newly emerged strategy for potential causal inference that could reduce bias due to confounding and reverse causality with genetic variants as instrumental variables (21). In the context of the current global pandemic, the retrospective design but prospective nature of MR undoubtedly has the potential to offer a unique perspective on the pathophysiology and targeted therapies of COVID-19 in a more flexible and inexpensive manner (22). In addition, due to the specificity of COVID-19, all the patients included in the analysis were clinically accurate diagnoses, which reduces the possible selection bias.

Therefore, in the present study, using bidirectional MR, we investigated the potential causal association between COVID-19 and psychiatric illnesses by severity, based on hospitalization and critical illness statistics. Based on a recent study with a large sample size, we chose four psychiatric illnesses that were reported to be COVID-19-related mental health problems: ADHD, schizophrenia, bipolar disorder, and depression (12).

## Methods

### Mental Illnesses

Summary statistics of four mental illnesses (schizophrenia, depression, bipolar disorder, and ADHD) were drawn from the publicly available genome-wide association study (GWAS) summary data sources on the MR-Base platform, which is available at https://elifesciences.org/articles/34408. Basic information on schizophrenia (33,640 cases vs. 43,456 control participants), depression (59,851 cases vs. 113,154 control participants), bipolar disorder (20,352 cases vs. 31,358 control participants), and ADHD (20,183 cases vs. 35,191 control participants) is shown in Table 1.

**Table 1:** Psychiatry diseases and COVID-19 genetic summary data sources.

**Trait**	**Sample_size**	**ncase**	**ncontrol**	**Population**
ADHD (23)	55,374	20,183	35,191	European
Schizophrenia (24)	77,096	33,640	43,456	European
Bipolar disorder (25)	51,710	20,352	31,358	European
Major depressive disorder (26)	173,005	59,851	113,154	European
Critically ill COVID-19^*^	707,407	4,606	702,801	European
Hospitalized COVID-19^*^	1,206,629	9,373	1,197,256	European

* *Available from: https://www.covid19hg.org*.

Genetic variants that passed uncorrelated (*r*^2^ < 0.001) single-nucleotide polymorphisms (SNPs) associated with the risk factor at thresholds for a genome-wide level of statistical significance (*p* < 5 × 10^−8^) were selected as instruments.

### COVID-19

We drew on summary statistics for critically ill COVID-19 cases and hospitalized COVID-19 cases from release five (https://www.covid19hg.org/results/r5/) of the COVID-19 Host Genetics Initiative Genome-Wide Association Study, which contained 4,606 critically ill COVID-19 cases and 702,801 controls (leave out study: 23andMe) and 9,373 hospitalized COVID-19 cases and 1,197,256 controls (leave out study: 23andMe) (Table 1). Hospitalized COVID-19 cases were defined as those who had “laboratory-confirmed SARS-CoV-2 infection and were hospitalized for COVID-19.” Critically ill cases were defined as those who had “laboratory-confirmed SARS-CoV-2 infection and were hospitalized for COVID-19 (death or respiratory support)” (https://www.covid19hg.org/blog/2021-03-02-freeze-5-results/). Controls are defined as everyone who was not a case, e.g., the healthy population. These two phenotypes are referred to as A2 and B2 in the COVID-19 Host Genetics Initiative documentation, respectively. As described previously, we used independent clumped SNPs meeting a threshold (*r*^2^ < 0.001, *p* < 5 × 10^−8^) as instrumental variables.

### MR Analysis

Mendelian randomization analyses were conducted in R, version 4.0.3 (http://www.r-project.org) using the TwoSampleMR package.

For each direction of potential influence, three MR methods were conducted in the present study. Conventional inverse-variance weighting (IVW), the most powerful method, was conducted to estimate the association of genetically proxied levels of mental illness with the risk of critically ill COVID-19 and hospitalized COVID-19. In addition, analyses were bidirectional to assess reverse causality. To reduce bias caused by horizontal pleiotropy (27), which influences the outcomes through causal pathways rather than exposure, we conducted two other established MR methods, including the weighted median and MR-Egger regression methods. These two methods are relatively robust to horizontal pleiotropy at the expense of statistical power. MR-PRESSO (Pleiotropy Residual Sum and Outlier) was applied to detect widespread horizontal pleiotropy for all results (28). Effect estimates were converted to odds ratio (OR), as the outcome was dichotomous.

Finally, to assess the robustness of significant results, for statistically significant results, further tests for horizontal pleiotropy, including the MR-Egger intercept test of deviation from the null, leave-1-SNP-out analyses and the modified Cochran Q statistic, were conducted to detect heterogeneous outcomes (29). Two-tailed tests were used for all statistical tests. To account for multiple testing in our primary analyses of COVID-19 in relation to the four outcomes, a Bonferroni-corrected threshold of *p* < 0.013 (*a* = 0.05/4 outcomes) was used. Associations with *p*-values between 0.013 and 0.05 were considered suggestive evidence of associations, requiring further confirmation.

### Ethical Approval, Data Availability, and Reporting

We obtained summary data from published studies, which were approved by the institutional review committees in their respective studies. Therefore, no further sanction was required.

## Results

### Participants and Genetic Instrumental Variables for Mental Illnesses and COVID-19

There were 81, 5, 16, 12, 8, and 5 independent variants for schizophrenia, depression, bipolar disorder, ADHD, critically ill COVID-19, and hospitalized COVID-19, respectively. Genetic instruments for critically ill COVID-19 (leave out 23andme), hospitalized COVID-19 (leave out 23andme), schizophrenia, depression, bipolar disorder, and ADHD by each instrumental SNP (GWAS significance with *p* < 5 × 10^−8^ and linkage disequilibrium threshold with *r*^2^ < 0.001) are listed in Supplementary Tables 1–6.

### Effects of Mental Illnesses on COVID-19

**Figure 1:**
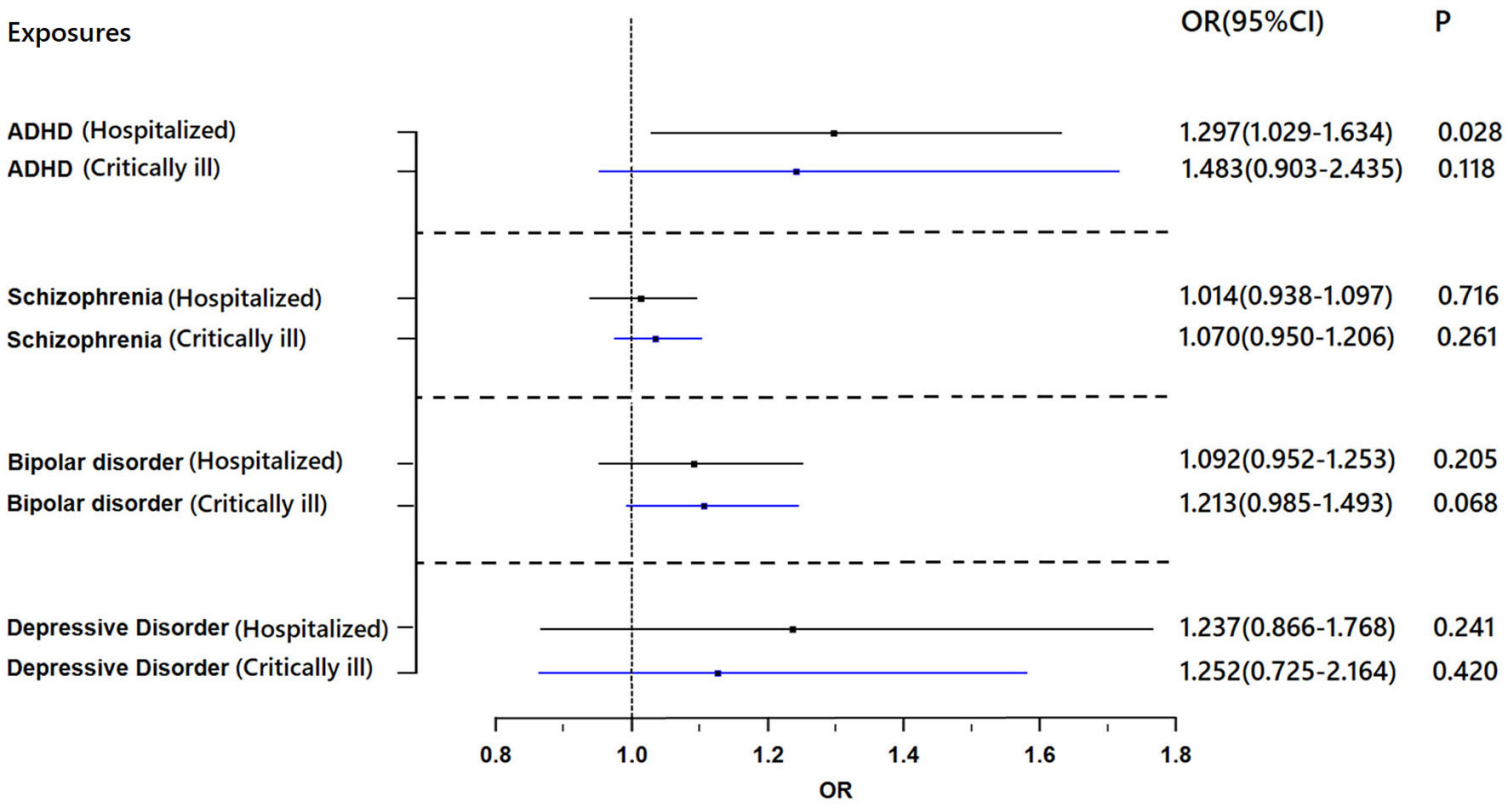
Results of the Mendelian randomization analysis investigating the association of genetically proxied mental illnesses with risk of hospitalized COVID-19 and critically ill COVID-19. Forest plot showing inverse-variance weighted Mendelian randomization estimates for the association between mental illnesses and risk of hospitalized COVID-19 (*n* = 1,388,342) and critically ill COVID-19 (*n* = 1,887,658). ADHD, attention-deficit/hyperactivity disorder; OR, odds ratio.

**Figure 2:**
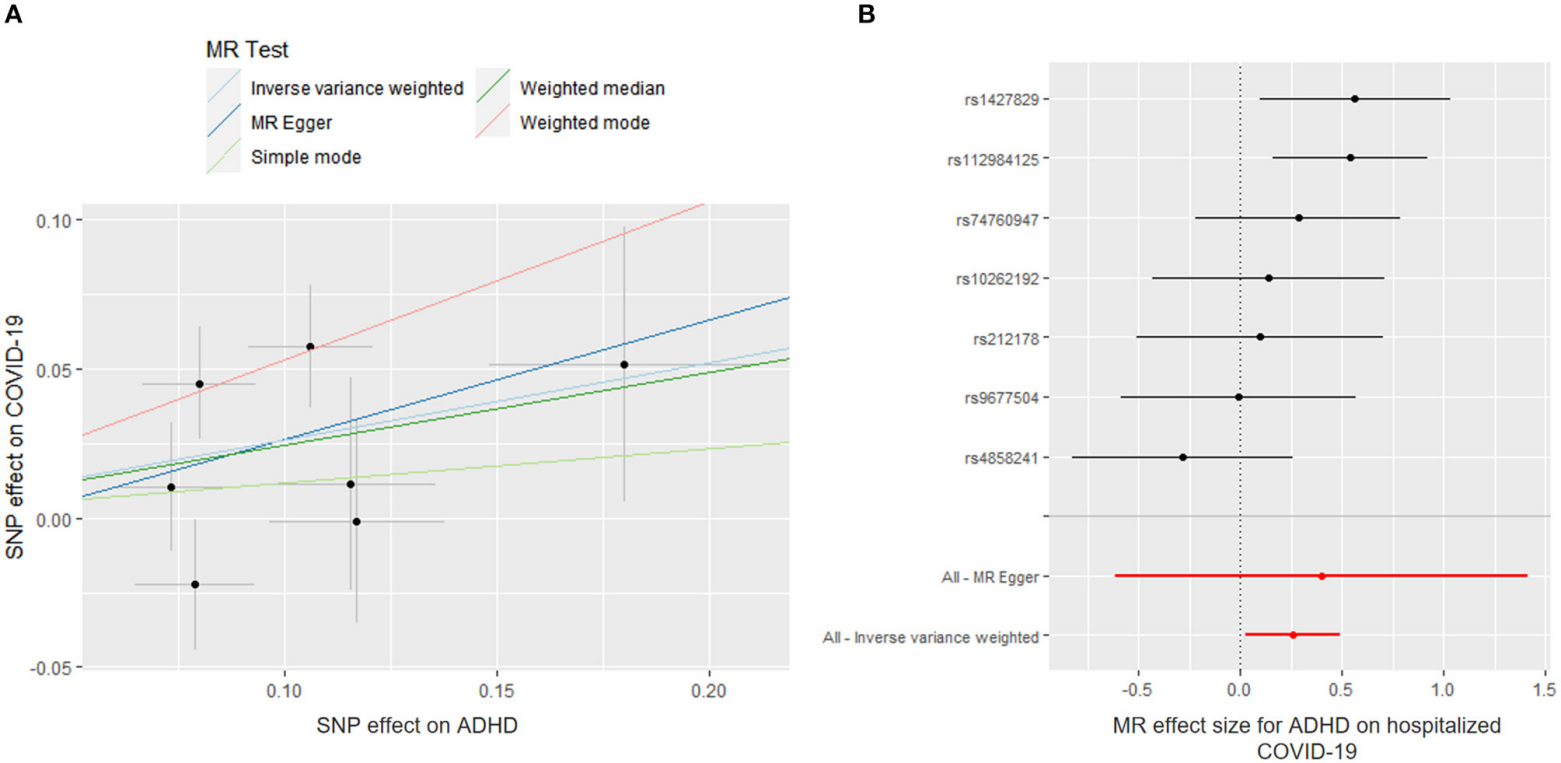
Mendelian randomization (MR) plots for relationship of ADHD with hospitalized COVID-19. **(A)** Scatter plot of single-nucleotide polymorphism (SNP) effects on ADHD vs. their effects on hospitalized COVID-19. The slope of each line indicated MR effect of every method. **(B)** Forest plot of causal effect size of each SNP on total hospitalized COVID-19 risk.

Figure 1 shows the association between genetically proxied mental illnesses and COVID-19. There is a suggestive causal association between genetically predicted ADHD and an increased risk of hospitalized COVID-19. The OR was 1.297 [95% confidence interval (CI), 1.029–1.634; *p* = 0.028]. A similar suggestive association was obtained in MR sensitivity analyses of weighted median (OR = 1.315; 95% CI, 1.007–1.717; *p* = 0.044); point estimates were consistent when using MR-Egger but produced 95% CIs that crossed the null (Supplementary Table 7). For the SNPs, MR-PRESSO did not detect any potential pleiotropy (*p* = 0.202). Moreover, leave-1-SNP-out analyses did not indicate that any SNP drove the result, but rather reflected an overall combined pattern of opposite relationships with ADHD and hospitalized COVID-19 (Figure 2). The modified Cochran Q statistic revealed no notable heterogeneity (*Q* = 8.942; *p* = 0.177) across instrument SNP effects. The result from the MR-Egger intercept test did not reveal any unbalanced horizontal pleiotropy (intercept *p*-value = 0.780).

However, no evidence of a directional causal relationship between ADHD and critically ill COVID-19 was found. Similarly, there was no evidence supporting an association of schizophrenia, depression, or bipolar disorder with the risk of critically ill COVID-19 or hospitalization with COVID-19.

### Effects of COVID-19 on Mental Illnesses

**Figure 3:**
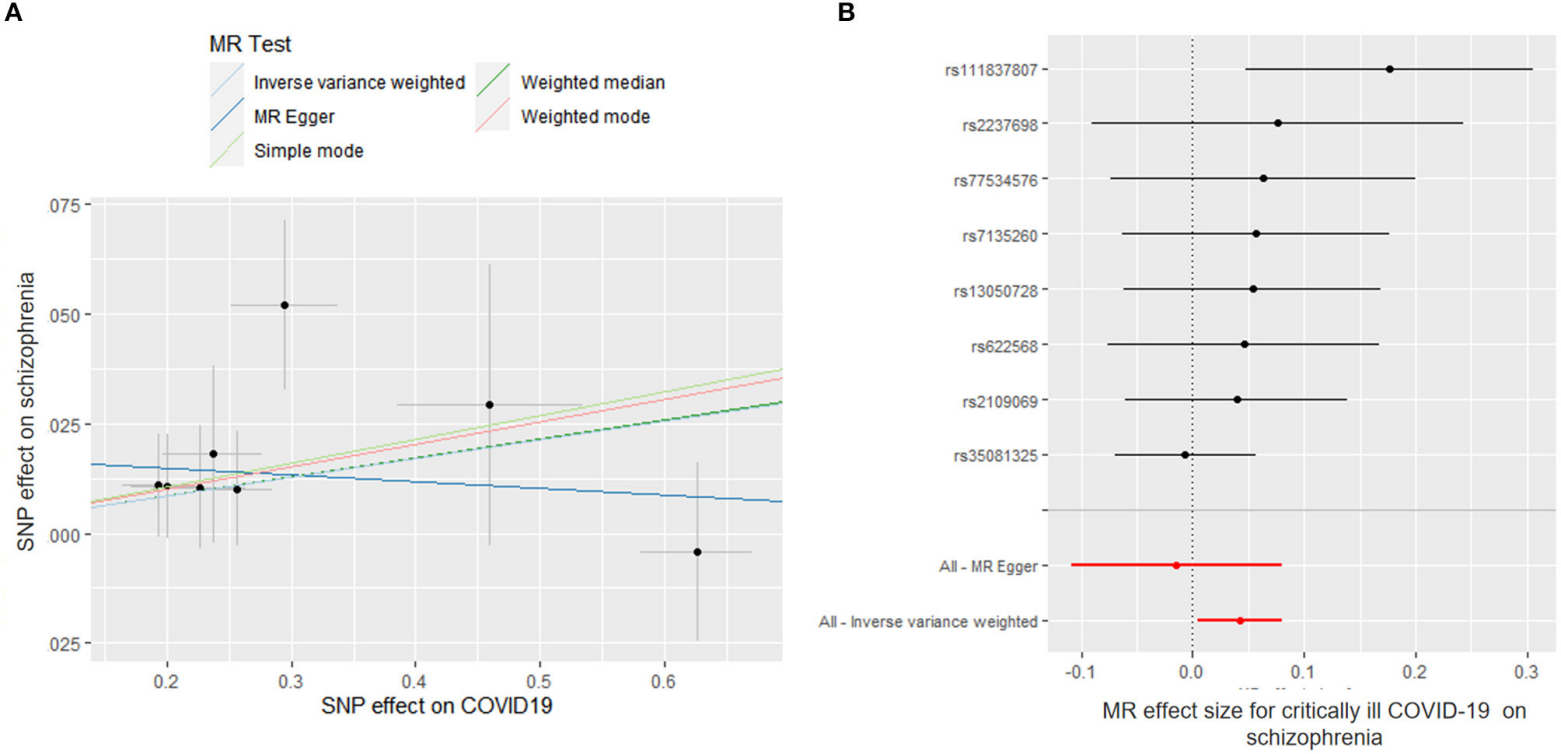
Mendelian randomization (MR) plots for relationship of critically ill COVID-19 with schizophrenia. **(A)** Scatter plot of single-nucleotide polymorphism (SNP) effects on critically ill COVID-19 vs. their effects on schizophrenia. The slope of each line indicated MR effect of every method. **(B)** Forest plot of causal effect size of each SNP on total schizophrenia risk.

We found a suggestive causal association between genetically predicted critically ill COVID-19 and higher odds of schizophrenia (OR = 1.043; 95% CI, 1.005–1.082; *p* = 0.027). Likewise, leave-1-SNP-out analyses and the modified Cochran Q statistic (*Q* = 6.810; *p* = 0.450) detected no heterogeneous outcomes. The MR-PRESSO test also showed no outlier pleiotropy (*p* = 0.413). However, there was no evidence of causal relationships of critically ill COVID-19 with schizophrenia across other MR methods (weighted median: OR = 1.045; 95% CI, 0.995–1.097; *p* = 0.083; MR-Egger: OR = 0.985; 95% CI, 0.897–1.082; *p* = 0.760) (Figure 3). The MR-Egger intercept test further suggested that there was no evidence of directional pleiotropy between hospitalized COVID-19 and schizophrenia (intercept *p*-value = 0.195).

**Figure 4:**
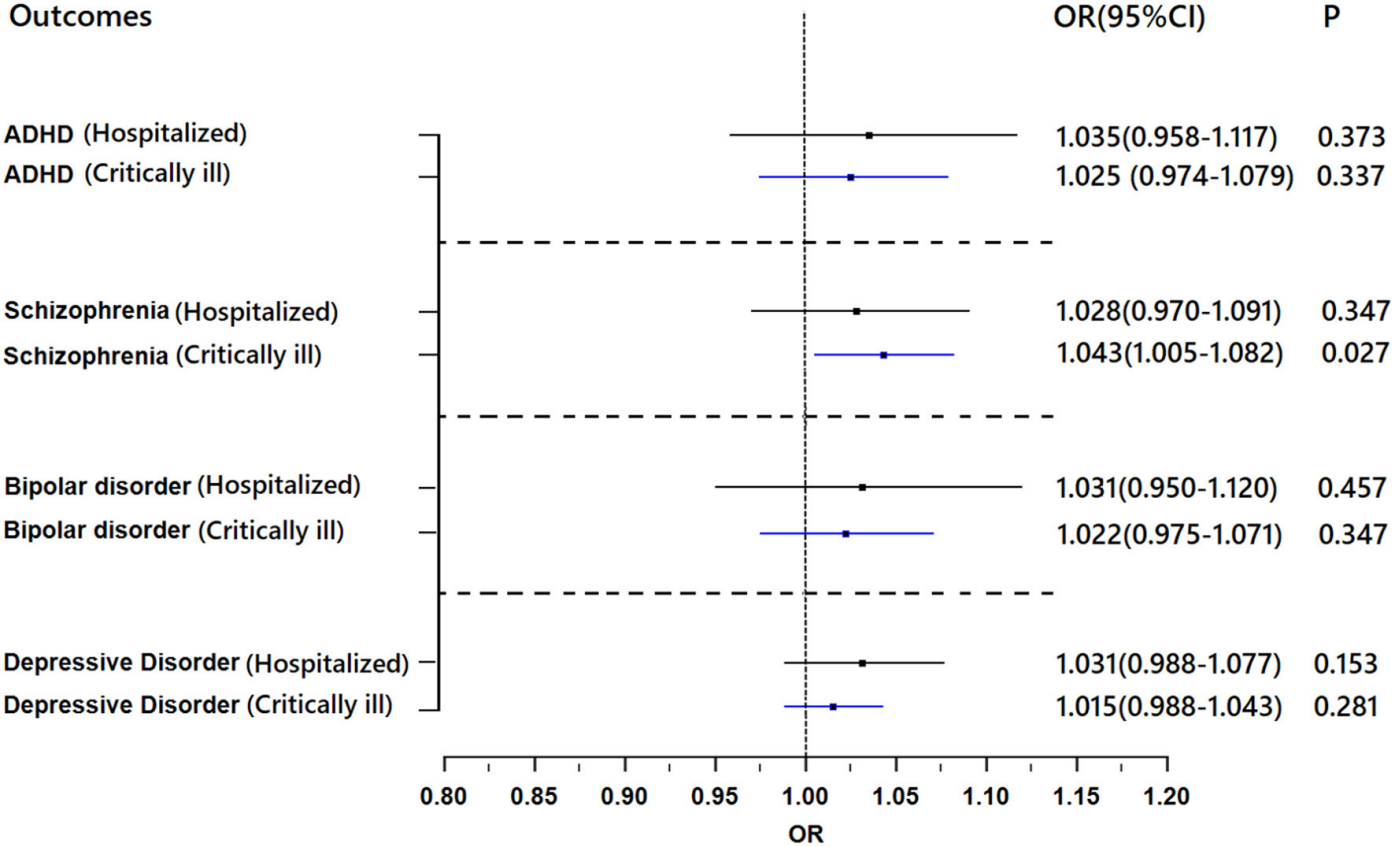
Results of the Mendelian randomization analysis investigating the association of genetically proxied COVID-19 with risk of mental illnesses. Forest plot showing inverse-variance weighted Mendelian randomization estimates for the association between COVID-19 and risk of ADHD (*n* = 55,374), schizophrenia (*n* = 77,096), bipolar disorder (*n* = 51,710), and major depressive disorder (*n* = 173,005). ADHD, attention-deficit/hyperactivity disorder; OR, odds ratio.

Genetically predicted critically ill COVID-19 and hospitalization with COVID-19 were not associated with depression, bipolar disorder or ADHD (Figure 4). For all results, the MR-Egger intercepts were approximately equal to 1.00 with narrow 95% CIs, which suggests no strong unbalanced horizontal pleiotropy (Supplementary Table 8).

## Discussion

In the present two-sample MR study, based on previous observational research, we systematically evaluated the associations of COVID-19 with four mental illnesses with publicly available large-scale GWAS datasets. We found that to a minor extent, ADHD was linked to increased odds of hospitalized COVID-19 and genetic predisposition to critical illness with COVID-19 was associated with significantly higher odds of schizophrenia.

### Effects of Mental Illnesses on COVID-19

A statistically significant positive association was observed with a genetic predisposition to ADHD and hospitalized COVID-19 in our study. Previous studies found that individuals with a diagnosis of ADHD had a higher risk for COVID-19 infection, which has been reported in some studies with large sample sizes (10, 12). Merzon et al. even found that only the rate of ADHD was significantly higher among COVID-19 subjects than among all the other mental health disorders that were assessed (10). The explanation has been attributed to specific manifestations of ADHD. For example, inattention, hyperactivity, and impulsivity might place such individuals at higher risk for forgetting to wash hands or wear masks in public, which could increase their probability of exposure to COVID-19. Here, we found evidence at the genetic level. Notably, Arbel et al. found that recovery rates of coronavirus increase with the prevalence of ADHD. Therefore, they proposed that ADHD might provide an evolutionary advantage, as with the natural immunity to malaria brought by sickle-cell disease. This might explain our findings that ADHD could lead to higher odds of hospitalized COVID-19 rather than critically ill COVID-19 (30). However, more studies are needed before a definitive answer is reached.

Although our study only found a small causal relationship between ADHD and hospitalized COVID-19 with large-scale GWAS, the characteristics of ADHD could interfere with their ability to comply with public health measures, which might further increase their vulnerability to being infected by COVID-19. This reminds us that enhanced protection and treatment of key populations, such as individuals with ADHD, is needed to reduce the spread of COVID-19 infection. It is worth noting that previous studies have proven that pharmacotherapy of ADHD might moderate the risk of infection (10). Moreover, vaccinations are performed to enhance immunity and reduce the risk of infection or infection-related death, while decisions on where and to whom to offer COVID-19 vaccinations as a priority are complex and need to consider estimated numbers of individuals at high risk of infection, cost, lack of quantities and other factors. Choosing high-risk diseases, such as ADHD, as a priority may be a logical approach to protect these patients from being infected by COVID-19.

### Effects of COVID-19 on Mental Illnesses

Since the COVID-19 outbreak in 2019, neuropsychiatric symptoms have been frequently reported in COVID-19 patients. Some scholars have proposed that COVID-19 is likely to have potential neuroinvasive and neurotropic capabilities and numerous mechanisms are likely to be involved in changes to mental state (31).

Schizophrenia has engendered some of the most intense skepticism in COVID-19. According to past pandemics and recent neurobiological evidence, researchers have long suspected that COVID-19 would present a significant risk for the development of schizophrenia. However, direct evidence of the association between COVID-19 and schizophrenia could not be provided. By MR, our study supports the causal association between COVID-19 and schizophrenia and provides a strong genetic instrument. These results resonate with observational studies. As early as the seventeenth and eighteenth centuries, nervous sequelae of infection were noted (32). A significant number of acute post-influenza psychoses were reported in the aftermath of the Spanish flu pandemic. Severe acute respiratory syndrome (SARS) survivors still showed elevated stress levels 1 year after the outbreak (33). Similarly, increased risks of both schizophrenia and acute psychosis have been described after HIV infection (34). Recently, one meta-analysis suggested that increasing serum interleukin-6 (IL-6) was associated with severe COVID-19 (35). Coincidentally, another study found that subjects with schizophrenia also had significantly higher peripheral and cerebrospinal fluid IL-6 than the controls (36). Therefore, it is not rigorous to establish a causal relationship between COVID-19 and schizophrenia.

This analysis provided evidence that critically ill COVID-19 infection may confer a long-term risk for psychosis. Although the increased risk is modest from the genetic perspective, the ongoing massive worldwide exposure to COVID-19 is likely to substantially increase the number of individuals diagnosed with schizophrenia. In addition, considering that negative environmental and psychosocial factors during COVID-19 itself are likely to exacerbate or induce mental illness, an explosion in the number of people with schizophrenia is therefore likely to be inevitable. Schizophrenia is a high-burden non-communicable condition associated with years of life lived with disability that could bring a heavy burden on patients and their families and challenge the global health system. Although the global prevalence of schizophrenia is only approximately 0.4% (37), the disease costs the world economy hundreds of billions of dollars per year (38). Therefore, we believe that there is a great need to pay attention to neuropsychiatric complications and the long-term mental effects of COVID-19, especially in severe patients. A systematic and practical clinical strategy and system should be built to counteract the possible outbreak of mental illness. More basic clinical research is needed to understand their influential factors and mechanisms to provide effective and accessible interventions to improve their quality of life.

## Limitations

In the present MR study, we included European ancestry populations to provide further evidence to support causal relationships between COVID-19 and mental illness and to some extent confirm previous speculations.

Our study should be evaluated in light of some shortcomings. First, our study was based on the European population; therefore, generalizability to other populations cannot be assumed. Second, although our results verify the causal relationships between COVID-19 and schizophrenia and ADHD, confirmation of causal effects may require more studies in the future. It is noteworthy that our estimates can explain the lifelong average effects of genetic variants. Therefore, our causal association cannot be fully interpreted in the same way, similar to the effects from an observational study or within a briefer-period observation. Moreover, even though no causal association was observed from an MR result, the potential importance of a factor may exist within shorter time frames (e.g., depression and bipolar disorder), and further investigation may be needed to find relevant discrepancies (39). Finally, this work analyzed only four diseases based on a large nationwide database study. There might be causal associations between other psychiatric diseases and COVID-19. However, it is still noteworthy that MR is an approach for testing causal hypotheses using genetic data from observational data (23, 24), which utilizes genetic variants of risk factors as instruments to assess its effect on particular disease, exploiting the random allocation of genetic variants to infer the causality. Therefore, not all mental illnesses were included in the study, such as autism spectrum disorder and anxiety.

## Conclusion

With MR, our study validates that a diagnosis of ADHD is associated with a higher risk of hospitalized COVID-19. In addition, we support the well-established relationship between critical COVID-19 and schizophrenia. Although the increased risk is marginal, given the characteristics of ADHD, which might further increase the affected individuals' vulnerability to being infected by COVID-19, the current SARS-CoV-2 pandemic and the high burden of schizophrenia, our work implies that enhancing ADHD-related therapeutic and preventive actions to reduce infection rates of hospitalized COVID-19 patients, examining psychiatric sequelae of critically ill COVID-19 patients, and building a systematic and practical clinical strategy to counteract the possible outbreak of mental illness will be important in the future.

## Data Availability Statement

The original contributions presented in the study are included in the article/Supplementary Material, further inquiries can be directed to the corresponding authors.

## Author Contributions

NL: preparation, creation, and/or presentation of the published work, specifically writing the initial draft (including substantive translation). J-ST: application of statistical, mathematical, computational, or other formal techniques to analyze or synthesize study data. LL: ideas, formulation or evolution of overarching research goals and aims. YW: management and coordination responsibility for the research activity planning and execution. LH: acquisition of the financial support for the project leading to this publication. QQ: preparation, creation, and/or presentation of the published work by those from the original research group, specifically critical review, commentary, or revision—including pre- or post-publication stages. All authors contributed to the article and approved the submitted version.

## Funding

This work was supported by the National Science Foundation of China (81571340 and 81873802), the Capita's Funds for Health Improvement and Research (CFH: 2020-2-4112), the National Key Basic Research Program of China (973 program 2014CB846104), and the CAMS Innovation Fund for Medical Sciences (CIFMS) (ID 2017-I2M-3-003), National Clinical Research Center for Cardiovascular Diseases, Fuwai Hospital, Chinese Academy of Medical Sciences (NCRC2020007), and National Clinical Research Center for Cardiovascular Diseases, Fuwai Hospital, Chinese Academy of Medical Sciences (NCRC2020007).

## Conflict of Interest

The authors declare that the research was conducted in the absence of any commercial or financial relationships that could be construed as a potential conflict of interest.

## Publisher's Note

All claims expressed in this article are solely those of the authors and do not necessarily represent those of their affiliated organizations, or those of the publisher, the editors and the reviewers. Any product that may be evaluated in this article, or claim that may be made by its manufacturer, is not guaranteed or endorsed by the publisher.
