# Corrigendum: Genetic predisposition between COVID-19 and four mental illnesses: A bidirectional, two-sample Mendelian randomization study

**DOI:** 10.3389/fpsyt.2022.1070770

**Published:** 2022-11-04

**Authors:** Ningning Liu, Jiang-Shan Tan, Lu Liu, Yufeng Wang, Lu Hua, Qiujin Qian

**Affiliations:** ^1^Peking University Sixth Hospital/Institute of Mental Health, Beijing, China; ^2^NHC Key Laboratory of Mental Health (Peking University), National Clinical Research Center for Mental Disorders (Peking University Sixth Hospital), Beijing, China; ^3^Thrombosis Center, Key Laboratory of Pulmonary Vascular Medicine, National Clinical Research Center of Cardiovascular Diseases, State Key Laboratory of Cardiovascular Disease and Fuwai Hospital, Chinese Academy of Medical Sciences and Peking Union Medical College, Beijing, China

**Keywords:** COVID-19, mental illness, GWAS, risk, Mendelian randomization

In the published article, there was a statistical error. The sample sizes was described as “We drew on summary statistics for critically ill COVID-19 cases and hospitalized COVID-19 cases from release five (https://www.covid19hg.org/results/r5/) of the COVID-19 Host Genetics Initiative Genome-Wide Association Study, which contained 5,101 critically ill COVID-19 cases and 1,383,241 controls (leave out study: 23andMe) and 9,986 hospitalized COVID-19 cases and 1,877,672 controls (leave out study: 23andMe) (Table 1).”

**Table 1:** Psychiatry diseases and COVID-19 genetic summary data sources.

**Trait**	**Sample_size**	**ncase**	**ncontrol**	**Population**
ADHD (23)	55,374	20,183	35,191	European
Schizophrenia (24)	77,096	33,640	43,456	European
Bipolar disorder (25)	51,710	20,352	31,358	European
Major depressive disorder (26)	173,005	59,851	113,154	European
Critically ill COVID-19^*^	707,407	4,606	702,801	European
Hospitalized COVID-19^*^	1,206,629	9,373	1,197,256	European

* Available from: https://www.covid19hg.org.

A correction has been made to **Methods**, “COVID-19,” Paragraph 1.

The corrected sentence appears below:

“We drew on summary statistics for critically ill COVID-19 cases and hospitalized COVID-19 cases from release five (https://www.covid19hg.org/results/r5/) of the COVID-19 Host Genetics Initiative Genome-Wide Association Study, which contained 4,606 critically ill COVID-19 cases and 702,801 controls (leave out study: 23andMe) and 9,373 hospitalized COVID-19 cases and 1,197,256 controls (leave out study: 23andMe) (Table 1).”

In the published article, there was an error in Table 1 as published. The sample size of critically ill COVID-19 and hospitalized COVID-19 was incorrect. The corrected Table 1 and its caption appear below.

The authors apologize for these errors and state that this does not change the scientific conclusions of the article in any way. The original article has been updated.

## Publisher's note

All claims expressed in this article are solely those of the authors and do not necessarily represent those of their affiliated organizations, or those of the publisher, the editors and the reviewers. Any product that may be evaluated in this article, or claim that may be made by its manufacturer, is not guaranteed or endorsed by the publisher.

