# Supplementary material for: Genetic Predisposition Between COVID-19 and Four Mental Illnesses: A Bidirectional, Two-Sample Mendelian Randomization Study
Source: Front Psychiatry. 2021 Oct 20;12:746276. doi: 10.3389/fpsyt.2021.746276 (PMC8564104; doi:10.3389/fpsyt.2021.746276)
Supplement: Supplementary file 1 [file Data_Sheet_1.DOCX]

**Supplementary material online**

**Table S1.**  List of Genetic Instruments for schizophrenia by Each Instrumental SNPs (GWAS Significance with P < 5×10^−8^ and linkage disequilibrium threshold with R^2^ < 0.001)

| **No.** | **SNP** | **Chr.** | **EA** | **OA** | **EAF** | **β** | **SE** |
| --- | --- | --- | --- | --- | --- | --- | --- |
| 1 | rs35998080 | 1 | T | G | 0.45 | 0.069004 | 0.0112 |
| 2 | rs4648845 | 1 | T | C | 0.479 | 0.067201 | 0.0119 |
| 3 | rs1702294 | 1 | C | T | 0.784 | 0.118401 | 0.0138 |
| 4 | rs12062861 | 1 | A | G | 0.175 | -0.091096 | 0.0149 |
| 5 | rs301797 | 1 | A | C | 0.322 | 0.066097 | 0.0116 |
| 6 | rs1498232 | 1 | C | T | 0.697 | -0.072004 | 0.0118 |
| 7 | rs11210892 | 1 | A | G | 0.652 | -0.067797 | 0.0115 |
| 8 | rs77149735 | 1 | A | G | 0.016 | 0.284502 | 0.0485 |
| 9 | rs11682175 | 2 | C | T | 0.439 | 0.073496 | 0.0109 |
| 10 | rs55775495 | 2 | T | C | 0.701 | -0.068996 | 0.0114 |
| 11 | rs11693094 | 2 | T | C | 0.429 | -0.073603 | 0.011 |
| 12 | rs11693528 | 2 | G | C | 0.185 | 0.102797 | 0.0136 |
| 13 | rs76355118 | 2 | G | A | 0.041 | 0.154399 | 0.0278 |
| 14 | rs2909457 | 2 | A | G | 0.574 | -0.059697 | 0.0109 |
| 15 | rs6704768 | 2 | A | G | 0.529 | -0.0766 | 0.0109 |
| 16 | rs1509378 | 2 | G | A | 0.646 | -0.0692 | 0.0119 |
| 17 | rs7601312 | 2 | G | A | 0.484 | 0.058997 | 0.0108 |
| 18 | rs75968099 | 3 | T | C | 0.351 | 0.080104 | 0.0114 |
| 19 | rs2535627 | 3 | C | T | 0.503 | -0.070403 | 0.0109 |
| 20 | rs34796896 | 3 | A | G | 0.194 | -0.082198 | 0.0135 |
| 21 | rs17194490 | 3 | T | G | 0.165 | 0.0966 | 0.0148 |
| 22 | rs832190 | 3 | T | C | 0.613 | -0.069897 | 0.0113 |
| 23 | rs6439649 | 3 | T | G | 0.562 | 0.070999 | 0.0111 |
| 24 | rs1106568 | 4 | A | G | 0.744 | -0.069404 | 0.0125 |
| 25 | rs215411 | 4 | A | T | 0.327 | 0.0692 | 0.0115 |
| 26 | rs35225200 | 4 | C | A | 0.087 | 0.144795 | 0.0203 |
| 27 | rs4391122 | 5 | G | A | 0.462 | 0.078005 | 0.0109 |
| 28 | rs3849046 | 5 | T | C | 0.508 | 0.062496 | 0.0109 |
| 29 | rs111294930 | 5 | G | A | 0.219 | -0.087699 | 0.0143 |
| 30 | rs11740474 | 5 | T | A | 0.412 | 0.062695 | 0.0112 |
| 31 | rs16867576 | 5 | G | A | 0.12 | -0.095801 | 0.017 |
| 32 | rs113397282 | 6 | C | T | 0.153 | -0.145 | 0.0176 |
| 33 | rs1233578 | 6 | G | A | 0.149 | -0.196397 | 0.0164 |
| 34 | rs3798869 | 6 | A | G | 0.464 | -0.066802 | 0.011 |
| 35 | rs186545906 | 6 | A | G | 0.108 | -0.118795 | 0.0196 |
| 36 | rs1339227 | 6 | T | C | 0.355 | -0.063302 | 0.0114 |
| 37 | rs117074560 | 6 | T | C | 0.037 | -0.156595 | 0.0277 |
| 38 | rs58120505 | 7 | C | T | 0.412 | -0.082197 | 0.0111 |
| 39 | rs12704290 | 7 | A | G | 0.131 | -0.106105 | 0.0168 |
| 40 | rs13240464 | 7 | C | T | 0.379 | -0.080704 | 0.0116 |
| 41 | rs7801375 | 7 | G | A | 0.847 | 0.083001 | 0.015 |
| 42 | rs36068923 | 8 | G | A | 0.187 | 0.083501 | 0.0134 |
| 43 | rs10108725 | 8 | T | C | 0.222 | 0.073204 | 0.0133 |
| 44 | rs4129585 | 8 | C | A | 0.558 | -0.079301 | 0.0109 |
| 45 | rs13261481 | 8 | G | T | 0.591 | -0.062402 | 0.011 |
| 46 | rs73191547 | 8 | T | A | 0.286 | 0.066899 | 0.0115 |
| 47 | rs73229090 | 8 | A | C | 0.121 | -0.0995 | 0.0177 |
| 48 | rs7819570 | 8 | T | G | 0.207 | 0.076498 | 0.014 |
| 49 | rs11139497 | 9 | A | T | 0.336 | 0.0656 | 0.0118 |
| 50 | rs11191419 | 10 | A | T | 0.334 | -0.101601 | 0.0118 |
| 51 | rs7893279 | 10 | G | T | 0.125 | -0.1124 | 0.0175 |
| 52 | rs11027857 | 11 | A | G | 0.587 | 0.063998 | 0.0109 |
| 53 | rs2514218 | 11 | T | C | 0.327 | -0.072205 | 0.0116 |
| 54 | rs73036062 | 11 | A | G | 0.21 | -0.089104 | 0.0135 |
| 55 | rs35324223 | 11 | G | A | 0.181 | 0.091995 | 0.0145 |
| 56 | rs10791097 | 11 | G | T | 0.52 | -0.0766 | 0.0109 |
| 57 | rs12826178 | 12 | T | G | 0.073 | -0.168206 | 0.0244 |
| 58 | rs1024582 | 12 | G | A | 0.66 | -0.098904 | 0.0115 |
| 59 | rs4766428 | 12 | T | C | 0.459 | 0.069395 | 0.0112 |
| 60 | rs1615350 | 12 | T | C | 0.734 | -0.0851 | 0.0123 |
| 61 | rs1191551 | 14 | G | T | 0.784 | -0.071697 | 0.0131 |
| 62 | rs12887734 | 14 | T | G | 0.273 | 0.088304 | 0.0121 |
| 63 | rs2693698 | 14 | G | A | 0.551 | 0.061705 | 0.0111 |
| 64 | rs17108967 | 14 | C | T | 0.343 | 0.065296 | 0.0115 |
| 65 | rs2332700 | 14 | G | C | 0.756 | -0.0771 | 0.0125 |
| 66 | rs2414718 | 15 | A | G | 0.617 | 0.069797 | 0.011 |
| 67 | rs28681284 | 15 | T | C | 0.214 | -0.101601 | 0.0141 |
| 68 | rs783540 | 15 | G | A | 0.43 | 0.059899 | 0.011 |
| 69 | rs4702 | 15 | A | G | 0.574 | -0.080505 | 0.0115 |
| 70 | rs12691307 | 16 | G | A | 0.478 | -0.071902 | 0.0113 |
| 71 | rs8055219 | 16 | A | G | 0.259 | 0.076998 | 0.0127 |
| 72 | rs12932476 | 16 | G | C | 0.512 | -0.059702 | 0.0109 |
| 73 | rs4523957 | 17 | T | G | 0.603 | 0.069703 | 0.0115 |
| 74 | rs11658257 | 17 | C | G | 0.573 | -0.066204 | 0.0115 |
| 75 | rs9636107 | 18 | G | A | 0.497 | 0.079595 | 0.0108 |
| 76 | rs72986630 | 19 | T | C | 0.054 | 0.1459 | 0.0266 |
| 77 | rs2905432 | 19 | A | G | 0.671 | -0.066001 | 0.0114 |
| 78 | rs2053079 | 19 | G | A | 0.265 | 0.071797 | 0.0127 |
| 79 | rs2103655 | 20 | A | G | 0.664 | 0.0766 | 0.0119 |
| 80 | rs5995756 | 22 | C | T | 0.54 | -0.072497 | 0.0109 |
| 81 | rs760648 | 22 | A | G | 0.416 | 0.075803 | 0.0118 |

**Table S2.**  List of Genetic Instruments for depression by Each Instrumental SNPs (GWAS Significance with P < 5×10^−8^ and linkage disequilibrium threshold with R^2^ < 0.001)

| **No.** | **SNP** | **Chr.** | **EA** | **OA** | **EAF** | **β** | **SE** |
| --- | --- | --- | --- | --- | --- | --- | --- |
| 1 | rs7531118 | 1 | C | T | NA | 0.0449974 | 0.008 |
| 2 | rs12129573 | 1 | A | C | NA | 0.0477992 | 0.0082 |
| 3 | rs76025409 | 5 | C | G | NA | 0.0567016 | 0.0085 |
| 4 | rs6905391 | 6 | A | G | NA | -0.0740018 | 0.0112 |
| 5 | rs1950829 | 14 | G | A | NA | -0.0454035 | 0.0079 |

**Table S3.**  List of Genetic Instruments for bipolar disorder by Each Instrumental SNPs (GWAS Significance with P < 5×10^−8^ and linkage disequilibrium threshold with R^2^ < 0.001)

| **No.** | **SNP** | **Chr.** | **EA** | **OA** | **EAF** | **β** | **SE** |
| --- | --- | --- | --- | --- | --- | --- | --- |
| 1 | rs2314398 | 2 | G | C | 0.319554 | -0.084102 | 0.0144 |
| 2 | rs9834970 | 3 | C | T | 0.501233 | 0.101003 | 0.0134 |
| 3 | rs2071044 | 3 | T | C | 0.467916 | -0.077702 | 0.0135 |
| 4 | rs11724116 | 4 | T | C | 0.155277 | -0.104095 | 0.0188 |
| 5 | rs329319 | 5 | G | A | 0.565703 | -0.078802 | 0.0139 |
| 6 | rs55648125 | 6 | G | A | 0.108936 | 0.117096 | 0.0215 |
| 7 | rs10455979 | 6 | G | C | 0.473478 | 0.075004 | 0.0137 |
| 8 | rs17150022 | 7 | C | T | 0.120723 | 0.113202 | 0.0204 |
| 9 | rs13231398 | 7 | C | G | 0.107064 | -0.1207 | 0.0219 |
| 10 | rs73496688 | 11 | A | T | 0.149117 | 0.108702 | 0.019 |
| 11 | rs174592 | 11 | G | A | 0.372297 | 0.0774 | 0.0141 |
| 12 | rs10744560 | 12 | T | C | 0.342297 | 0.083201 | 0.014 |
| 13 | rs71395455 | 15 | G | A | 0.31149 | -0.082096 | 0.0146 |
| 14 | rs884301 | 17 | T | C | 0.381084 | 0.080298 | 0.0138 |
| 15 | rs111444407 | 19 | T | C | 0.154691 | 0.1166 | 0.0184 |
| 16 | rs5758065 | 22 | G | C | 0.489522 | -0.074402 | 0.0135 |

**Table S4.**  List of Genetic Instruments for ADHD by Each Instrumental SNPs (GWAS Significance with P < 5×10^−8^ and linkage disequilibrium threshold with R^2^ < 0.001)

| **No.** | **SNP** | **Chr.** | **EA** | **OA** | **EAF** | **β** | **SE** |
| --- | --- | --- | --- | --- | --- | --- | --- |
| 1 | rs112984125 | 1 | A | G | NA | -0.106005 | 0.0146 |
| 2 | rs1222063 | 1 | A | G | NA | 0.0962007 | 0.0174 |
| 3 | rs9677504 | 2 | A | G | NA | 0.116903 | 0.0206 |
| 4 | rs4858241 | 3 | G | T | NA | -0.0789036 | 0.014 |
| 5 | rs28411770 | 4 | C | T | NA | -0.0861043 | 0.0151 |
| 6 | rs4916723 | 5 | C | A | NA | 0.0766003 | 0.0135 |
| 7 | rs10262192 | 7 | A | G | NA | 0.073204 | 0.0132 |
| 8 | rs74760947 | 8 | G | A | NA | 0.179797 | 0.0317 |
| 9 | rs11591402 | 10 | A | T | NA | -0.0929051 | 0.0164 |
| 10 | rs1427829 | 12 | G | A | NA | -0.0799012 | 0.0133 |
| 11 | rs281324 | 15 | C | T | NA | 0.0744973 | 0.0134 |
| 12 | rs212178 | 16 | A | G | NA | -0.1154 | 0.02 |

**Table S5.**  List of Genetic Instruments for critically ill COVID19 (leave 23andme) by Each Instrumental SNPs (GWAS Significance with P < 5×10^-8^ and linkage disequilibrium threshold with R^2^ < 0.001)

| **No.** | **SNP** | **Chr.** | **EA** | **OA** | **EAF** | **β** | **SE** |
| --- | --- | --- | --- | --- | --- | --- | --- |
| 1 | rs13050728 | 21 | C | T | 0.6627 | -0.20011 | 0.028559 |
| 2 | rs2109069 | 19 | A | G | 0.3287 | 0.25663 | 0.02807 |
| 3 | rs77534576 | 17 | T | C | 0.03465 | 0.45975 | 0.074941 |
| 4 | rs7135260 | 12 | C | T | 0.6742 | 0.19247 | 0.027983 |
| 5 | rs2237698 | 7 | T | C | 0.08971 | 0.23662 | 0.039653 |
| 6 | rs622568 | 7 | C | A | 0.1463 | 0.22629 | 0.037082 |
| 7 | rs111837807 | 6 | C | T | 0.0996 | 0.29453 | 0.04276 |
| 8 | rs35081325 | 3 | T | A | 0.07529 | 0.62617 | 0.044502 |

Chr. indicates chromosome; EA, effect allele; OA, other allele; EAF, effect allele frequency.

**Table S6.**  List of Genetic Instruments for hospitalized COVID19 (leave 23andme) by Each Instrumental SNPs (GWAS Significance with P < 5×10^−8^ and linkage disequilibrium threshold with R^2^ < 0.001)

| **No.** | **SNP** | **Chr.** | **EA** | **OA** | **EAF** | **β** | **SE** |
| --- | --- | --- | --- | --- | --- | --- | --- |
| 1 | rs13050728 | 21 | C | T | 0.6528 | -0.16832 | 0.020183 |
| 2 | rs2109069 | 19 | A | G | 0.3227 | 0.15131 | 0.019906 |
| 3 | rs2660 | 12 | A | G | 0.6902 | 0.11639 | 0.019406 |
| 4 | rs622568 | 7 | C | A | 0.1512 | 0.15355 | 0.026026 |
| 5 | rs35081325 | 3 | T | A | 0.08122 | 0.48825 | 0.031508 |

Chr. indicates chromosome; EA, effect allele; OA, other allele; EAF, effect allele frequency.

**Table S7.** Associations between genetically predicted COVID-19 and mental diseases in sensitivity analyses using the weighted median and MR-Egger methods

|  | **Weighted median** | |  | **MR-Egger** | | | |  |
| --- | --- | --- | --- | --- | --- | --- | --- | --- |
| **Outcome** | **OR (95% CI)** | ***P* value** |  | **OR (95% CI)** | ***P* value** |  | **Intercept^a^** | ***P* value** |
| **Critically ill COVID19 (leave 23andme)** |  |  |  |  |  |  |  |  |
| ADHD | 1.733(1.133-2.652) | 0.011 |  | 2.918(0.356-23.905) | 0.319 |  | 0.935(0.764-1.144) | 0.515 |
| Schizophrenia | 0.984(0.830-1.167) | 0.852 |  | 1.041(0.670-1.618) | 0.859 |  | 1.002(0.965-1.040) | 0.896 |
| Bipolar disorder | 1.302(0.988-1.717) | 0.062 |  | 1.719(0.511-5.785) | 0.381 |  | 0.967(0.861-1.085) | 0.566 |
| Major Depressive Disorder | 1.123(0.566-2.230) | 0.740 |  | 0.820(0.046-14.747) | 0.893 |  | 1.023(0.876-1.195) | 0.770 |
| **Hospitalized COVID19 (leave 23andme)** |  |  |  |  |  |  |  |  |
| ADHD | 1.315(1.007-1.717) | 0.044 |  | 1.493(0.540-4.130) | 0.441 |  | 0.986(0.894-1.088) | 0.780 |
| Schizophrenia | 0.9540(0.860-1.059) | 0.376 |  | 0.989(0.743-1.317) | 0.940 |  | 1.002(0.979-1.026) | 0.856 |
| Bipolar disorder | 1.070(0.892-1.284) | 0.465 |  | 1.523(0.692-3.356) | 0.295 |  | 0.969(0.899-1.043) | 0.401 |
| Major Depressive Disorder | 1.210(0.764-1.919) | 0.416 |  | 0.305(0.044-2.101) | 0.228 |  | 1.078(0.973-1.194) | 0.148 |

CI, confidence interval; OR odds ratio.

^a^ The MR-Egger intercept quantifies the effect of directional pleiotropy. Values that significantly differ from zero provide evidence that the COVID-19-associated single-nucleotide polymorphisms may influence the outcome through other pathways than through COVID-19.

**Table S8.** Associations between genetically predicted mental diseases and COVID-19 in sensitivity analyses using the weighted median and MR-Egger methods

|  | **Weighted median** | |  | **MR-Egger** | | | |  |
| --- | --- | --- | --- | --- | --- | --- | --- | --- |
| **Outcome** | **OR (95% CI)** | ***P* value** |  | **OR (95% CI)** | ***P* value** |  | **Intercept^a^** | ***P* value** |
| **Critically ill COVID19** **(leave 23andme)** |  |  |  |  |  |  |  |  |
| ADHD | 1.021(0.959-1.087) | 0.509 |  | 1.006(0.896-1.129) | 0.922 |  | 1.006(0.973-1.040) | 0.716 |
| Schizophrenia | 1.045(0.995-1.097) | 0.083 |  | 0.985(0.897-1.082) | 0.760 |  | 1.018(0.991-1.046) | 0.195 |
| Bipolar disorder | 0.996(0.937-1.058) | 0.905 |  | 0.951(0.844-1.072) | 0.412 |  | 1.022(0.989-1.057) | 0.195 |
| Major Depressive Disorder | 1.014(0.979-1.051) | 0.439 |  | 0.992(0.926-1.062) | 0.810 |  | 1.007(0.987-1.027) | 0.461 |
| **Hospitalized COVID19 (leave 23andme)** |  |  |  |  |  |  |  |  |
| ADHD | 1.033(0.947-1.126) | 0.472 |  | 1.025(0.883-1.190) | 0.745 |  | 1.002(0.971-1.034) | 0.881 |
| Schizophrenia | 1.044(0.975-1.118) | 0.222 |  | 0.965(0.856-1.087) | 0.550 |  | 1.015(0.992-1.039) | 0.226 |
| Bipolar disorder | 1.003(0.915-1.0998) | 0.941 |  | 0.969(0.811-1.159) | 0.737 |  | 1.014(0.979-1.051) | 0.441 |
| Major Depressive Disorder | 1.021(0.972-1.073) | 0.415 |  | 0.987(0.906-1.076) | 0.761 |  | 1.010(0.992-1.028) | 0.251 |

CI, confidence interval; OR odds ratio.

^a^ The MR-Egger intercept quantifies the effect of directional pleiotropy. Values that significantly differ from zero provide evidence that the COVID-19-associated single-nucleotide polymorphisms may influence the outcome through other pathways than through COVID-19.
